# Road building, land use and climate change: prospects for environmental governance in the Amazon

**DOI:** 10.1098/rstb.2007.0017

**Published:** 2008-02-11

**Authors:** Stephen Perz, Silvia Brilhante, Foster Brown, Marcellus Caldas, Santos Ikeda, Elsa Mendoza, Christine Overdevest, Vera Reis, Juan Fernando Reyes, Daniel Rojas, Marianne Schmink, Carlos Souza, Robert Walker

**Affiliations:** 1 Department of Sociology, University of FloridaPO Box 117330, Gainesville, FL 32611-7330, USA; 2 SOS Amazonia, Rua Pará 61, Cadeira VelhaRio Branco, Acre 69900-440, Brazil; 3 Woods Hole Research Center, 149 Woods Hole RoadFalmouth, MA 02540, USA; 4 Department of Geography, 118 Seaton Hall, Kansas State UniversityManhattan, KS 66506-2904, USA; 5 Proyecto Especial Madre de Dios, Instituto Nacional de DesarrolloJr San Martin S/W, Iberia, Madre de Dios, Peru; 6 Instituto de Pesquisa Ambiental da Amazônia, Avenida Nazaré 669Belém, Pará CEP 66035-170, Brazil; 7 Herencia, Calle Civica No. 47Barrio Miraflores, Cobija, Pando, Bolivia; 8 Universidad Amazónica de Pando, Calle Enrique Cornejo No. 77Cobija, Pando, Bolivia; 9 Center for Latin American Studies, University of FloridaPO Box 115530, Gainesville, FL 32611-5530, USA; 10 Instituto do Homem e Meio Ambiente da Amazônia, Rua Domingos Marreiros 2020Bairro Fátima, Belém, Pará CEP 66060-160, Brazil; 11 Department of Geography, Michigan State University, 116 Geography BuildingEast Lansing, MI 48824, USA

**Keywords:** Amazon, climate, deforestation, forests, governance, roads

## Abstract

Some coupled land–climate models predict a dieback of Amazon forest during the twenty-first century due to climate change, but human land use in the region has already reduced the forest cover. The causation behind land use is complex, and includes economic, institutional, political and demographic factors. Pre-eminent among these factors is road building, which facilitates human access to natural resources that beget forest fragmentation. While official government road projects have received considerable attention, unofficial road building by interest groups is expanding more rapidly, especially where official roads are being paved, yielding highly fragmented forest mosaics. Effective governance of natural resources in the Amazon requires a combination of state oversight and community participation in a ‘hybrid’ model of governance. The MAP Initiative in the southwestern Amazon provides an example of an innovative hybrid approach to environmental governance. It embodies a polycentric structure that includes government agencies, NGOs, universities and communities in a planning process that links scientific data to public deliberations in order to mitigate the effects of new infrastructure and climate change.

## 1. Introduction

While there is variation among coupled land–climate models as to prospects for the Amazon basin (Li *et al*. 2006), recent findings from the Hadley Centre model suggest a widespread forest dieback there in the twenty-first century (other articles, this volume). However, time-series data from satellite images confirm that deforestation due to human land use is already underway in the Amazon (INPE 2005) and will probably continue into the future (Laurance *et al*. 2001; Soares-Filho *et al*. 2006). Deforestation and forest fragmentation have negative ecological consequences (Bierregaard *et al*. 2001), which implies that Amazonia in the twenty-first century may be more vulnerable than climate models assume. This raises important questions about the drivers of forest clearing and fragmentation (Gutman *et al*. 2004; Lambin & Geist 2006), and how they operate in the Amazon (Perz 2002; Wood & Porro 2002).

In this paper, we focus on road building, a key determinant of land use in the Amazon. While road building exerts a key influence on forest fragmentation and therefore the ecological vulnerability of the Amazon, it is also instrumental for the viability of contemporary economic activity in the region and can cause social conflicts. Given these road impacts, we argue that innovative models of governance are needed to mitigate the negative socio-economic and ecological effects of roads, as well as the prospective impacts of climate change on the Amazon.

We first review theoretical explanations for deforestation, highlighting economic, institutional, political and demographic factors. We then focus on the issue of road building, particularly the construction of ‘unofficial roads’, which have received less attention than official infrastructure projects but which can generate considerable forest fragmentation and a litany of socio-economic and ecological consequences. A worrisome synergy in road building, namely that paving of official highways fosters the expansion of unofficial road networks, motivates a discussion of models of governance for roads and sustainable resource use. We consider state- and community-based models of governance, and suggest that each by itself is inadequate for road governance, which prompts articulation of a ‘hybrid’ state–community model. The paper concludes by discussing the case of the ‘MAP’ region in the southwestern Amazon, a tri-national frontier area that is incurring large-scale infrastructure investments for the Inter-Oceanic Highway. The ‘MAP Initiative’ there embodies a hybrid governance model by simultaneously seeking to mobilize civil society and engage governments in order to foment environmental governance across national boundaries. State–society engagement across such boundaries will be crucial to mitigate the impacts of new infrastructure as well as climate change.

## 2. Explanations for land use in the Amazon

Land use in the Amazon involves a variety of human activities and land users (Serrão & Homma 1993). Activities include extraction of timber and other forest products (such as rubber, castaña nuts, game, etc.), crop cultivation (whether annual crops such as rice and beans or perennial crops such as cocoa and coffee), cattle ranching (extensive or intensive) and mechanized agriculture (such as sugar cane and soya beans). Land users include a variety of social actors, ranging from indigenous peoples to colonist farm families, logging firms, large-scale ranchers and mechanized producers.

Pre-eminent among explanations for understanding land use are models that emphasize economic factors (Walker 2004). Micro-level land-use models focus on specific decision units, such as households, firms and communities, and emphasize the differences in their land, labour and capital as explanations for differences in deforestation and land use (Perz *et al*. 2006). In the Amazon, land is abundant relative to labour and capital, so social actors with more human and financial assets are better able to clear more forest. Economic perspectives on land use at the macro level emphasize market prices (Cattaneo 2002). Increases in land prices intensify land use, raising productivity per hectare and expanding land use farther from market centres. Conversely, rises in prices for agricultural inputs (such as labour or fertilizers) can restrict land use. The tendency in the Amazon has been for land prices to rise due to improved access to capital via new credit lines, favourable terms of trade for exports, and decreases in transport costs due to improved infrastructure.

Beyond strictly economic factors, many institutions influence land use. There has been considerable attention to land tenure, driven in part by longstanding debate over the necessity of secure private property rights as the best means to ensure sustainable land use (Ostrom 1990). In the Amazon, insecure tenure has in the past characterized areas experiencing rapid deforestation, as forest clearing in the absence of land titles is a means of demonstrating land claims (Alston *et al*. 1999). Recent years have seen a growing recognition of traditional tenure rights, most prominently in the case of extractive reserves, which are designed to maintain the forest cover while providing for forest-based livelihoods (Cavalcanti 2002; Ehringhaus 2006). Aside from land tenure, lending policies are important since they determine who gets bank credit, and tend to favour more capitalized producers. Similarly, state extension assistance can foster land use that avoids land degradation by disseminating information on new crop varieties and ‘best practices’ for agricultural technologies.

Political factors have also received considerable attention, especially due to instances of violence in conflicts over land in the Amazon (Wagner de Almeida 1995). The diversity of land users in the Amazon, and inequalities among them, helped foster the emergence of ‘political ecology’ as the study of contestation among social actors over the control of natural resources (Schmink & Wood 1992). In addition, political patronage, as in exchanges of votes for favours, has become important in local elections in the Amazon (Toni & Kaimowitz 2003), where such favours include road maintenance for supporters (Perz *et al*. 2007*a*). Political mobilization is increasingly common and takes a variety of forms across the basin, including formation of local organizations to improve product marketing, creation of networks for exchanges of labour or machinery and alliances to demand changes in state policies.

Explanations for land use continue to feature demographic factors. Malthusian arguments about population density and growth rates received limited empirical support with respect to deforestation in the Amazon (Perz 2002). More attention is now going to demographic dynamics at the household level. Households have life cycles, through which labour availability and child dependency change through family formation. Farm households with less dependency and more adult labourers tend to have larger production systems and more forest cleared (Perz *et al*. 2006).

There are still other determinants of land use and forest clearing, like technological change. Some have suggested that adoption of new technologies intensifies land use, reducing demand for more cleared land, but available evidence suggests that the opposite is often true in the Amazon (Angelsen & Kaimowitz 2001). There is also a longstanding discussion of the importance of cultural identity, whether defined in terms of place of birth (within or outside the Amazon), place attachment (and the desire to conserve forest as patrimony) or identification with specific natural resources (such as the culture of rubber tappers). Biophysical factors are also important, particularly soil fertility, topography, precipitation and agricultural pests (Schneider *et al*. 2000), as well as climatic variability and change (other papers, this volume).

## 3. Road building in the Amazon

In this paper, we focus on road building as a key determinant of land use. By reducing the costs of spatial mobility for people, capital and information, roads foster access to natural resources and facilitate market access for rural producers (Vance 1986; Owen 1987). However, roads fragment habitats, degrade stream networks and water quality, foster the spread of exotic invasive species, cause wildlife mortality and species loss, and may catalyse local climate change (Trombulak & Frissell 2000; Forman *et al*. 2003). In turn, habitat fragmentation and degradation leave forests more vulnerable to climate change. Roads can also cause social conflicts, via disputes over land and other natural resources, and by threatening habitats on which traditional livelihoods depend (Schmink & Wood 1992; Perz *et al*. 2005, 2007*a*). Econometric models confirm an independent effect of roads on deforestation in the Amazon (Andersen *et al*. 2002; Pfaff *et al*. 2007).

Because roads vary (whether paved or unpaved, etc.), it is important to draw distinctions among the types of roads. We emphasize the distinction between ‘official’ and ‘unofficial’ roads. Official roads are interregional highways built or financed by national or state governments. These highways appear on official maps and run hundreds of kilometres to link major cities. Official road projects in the Amazon constitute axes for continent-wide integration in South America, with the goal of facilitating resource exploitation for global markets to foster regional development (CEPEI 2002; IIRSA 2005). Examples include BR-163 through Pará and Mato Grosso (Brazilian states experiencing expansion of soya beans) and the Inter-Oceanic Highway (which links Atlantic ports in Brazil to Pacific ports in Peru). Such projects will probably increase deforestation in Amazonia (Laurance *et al*. 2001), though the extent of road impacts varies depending on the assumptions underlying land-use models (Soares-Filho *et al*. 2006). However, official roads form sparse networks, leaving large blocs of forest intact since parallel corridors are often hundreds of kilometres apart. Official roads have received the most attention, for the Amazon witnessed a previous generation of large-scale infrastructure projects that caused a widespread deforestation and social conflict (Goodland & Irwin 1975), which led to criticism and debate concerning contemporary projects (Laurance *et al*. 2001; Nepstad *et al*. 2002).

By contrast, unofficial roads are built by non-state social actors (Perz *et al*. 2005; Brandão *et al*. 2006). Unofficial roads merit more attention for several reasons. First, unofficial roads are specifically instrumental for natural resource exploitation (Perz *et al*. 2005, 2007*a*,*b*). Colonists and loggers build unofficial roads to gain access to land or timber, in order to support local livelihoods and community development. Second, unofficial roads are also called ‘endogenous roads’ because not only do they provide access to resources that support local livelihoods but those livelihoods also in turn provide funds to build more unofficial roads (Brandão *et al*. 2006; Pfaff *et al*. 2007). This feedback implies that so long as resource exploitation is profitable, unofficial road building will continue, even if it is not sustainable (Perz *et al*. 2005, 2007*b*). Third, unofficial roads form much denser networks in landscapes because they are often spaced only a few kilometres apart, frequently follow winding paths and have many intersections. Consequently, unofficial road networks fragment forest cover into smaller, more irregularly shaped and often more isolated patches that are more ecologically vulnerable. And finally, unofficial roads are expanding much more rapidly than official road networks in the Amazon. An analysis of satellite images of the centre-western portion of the Brazilian state of Pará revealed nearly a fourfold increase in unofficial roads in roughly a decade, such that by 2001, unofficial roads comprised over 80% of the total road network (Brandão *et al*. 2006). Unofficial roads also predominate in other portions of the Brazilian Amazon (Lentini *et al*. 2005, pp. 78–79).

Distinguishing between official and unofficial roads in the Amazon reveals an important synergy: paving of official roads motivates unofficial road building. Paving raises land values, which provides the incentive to exploit natural resources farther out from official road corridors. This in turn is made possible via construction or extension of unofficial roads, which then generate income that facilitates additional road building.

This synergy poses a dilemma for environmental governance in the Amazon (Perz *et al*. 2005, 2007*b*). On the one hand, official paving projects enjoy considerable political support and unofficial road building is crucial for local livelihoods. On the other hand, new infrastructure without environmental governance will probably lead to forest fragmentation and social conflicts. Such outcomes not only undermine the sustainability of current local livelihoods but also render forest more vulnerable to climate change, threatening future livelihood sustainability.

## 4. Models of governance and the case of road building

The dilemma of governing roads in the Amazon has prompted the discussion of new models of environmental governance (Perz *et al*. 2007*b*). Such discussions focus on questions of institutional design, that is, how best to formulate rules for access to and use of resources that involve transparency among stakeholders, effective but low-cost monitoring and graduated sanctions against violators (Ostrom 1990). Discussions of institutional design for resource governance focus on the state vis-á-vis other social actors. Consequently, there have been two predominant models of environmental governance: state- and community-based. However, both models face difficulties when applied to environmental governance in the Amazon due to the dilemma posed by road building (Perz *et al*. 2007*b*).

State-based perspectives emphasize national regulatory mechanisms such as creation of parks, tax breaks for sustainable resource use, restrictions such as quotas, and punitive measures for violations. Difficulties arise, however, to the extent that state agencies seek to implement uniform rules across localities with distinct circumstances, resulting in uneven implementation and unexpected outcomes (Perz *et al*. 2007*b*). Further, it is important to observe that economic recession in Brazil and other South American countries in the 1980s led to state withdrawal from investments in frontier expansion, which left local interest groups to their own devices. Local players now accustomed to relative autonomy in conducting business may resist externally imposed government interference in frontier areas of the Amazon. Such difficulties with state-based environmental governance may result in opposition and slow implementation (Perz *et al*. 2007*b*).

Liabilities of command-and-control approaches led to the advocacy of community-based governance models. Traditional people such as indigenous groups, riverine communities and forest-based extractivists have long-term experience and established institutions for regulating management of resources in the Amazon (Redford & Padoch 1992). Consequently, community-based proposals have emphasized community-level formulation and implementation of institutions for environmental governance based on local knowledge and traditional practice (Western & Wright 1994). However, community-based approaches are hampered by internal differences within communities that can cause conflicts or capture of governance by more powerful families, and because communities often have very limited capacity to deal with large-scale projects such as interregional highways (Perz *et al*. 2007*b*).

Such criticism led to a ‘new governance’ literature that emphasizes hybrid governance models which seek to combine the assets of state and community perspectives while avoiding their liabilities. Whereas the state has a greater capacity and the authority to impose discipline on local planning, communities are better able to include stakeholders in deliberations and design institutions to fit local realities (Perz *et al*. 2007*b*). In a hybrid model, the state provides public resources to support local governance. Communities formulate locally appropriate rules based on planning deliberations among stakeholders. State oversight ensures implementation, with the possibility for withdrawal of resources if local commitments are not being met. The economic importance of state funds and local roads serve as motivations for stakeholders to participate in a transparent fashion and for local players to watch each other, constituting a monitoring mechanism (Perz *et al*. 2007*b*). However, such hybrid models have not until recently been applied to environmental governance or the Amazon.

## 5. Experiments in environmental governance: the MAP Initiative

An experiment in hybrid approaches to environmental governance is underway in the southwestern Amazon, which faces daunting challenges to forest conservation due to infrastructure projects and climate change. As regards infrastructure, the final unpaved segments of the Inter-Oceanic Highway are being paved in the southwestern Amazon (IIRSA 2005). This project is being financed by the Andean Promotion Corporation (CAF) as well as the Brazilian and Peruvian governments, with a total budget exceeding US$890 million (Pro-Inversión 2005). Paving of the Inter-Oceanic Highway reduces transport costs in the southwestern Amazon and catalyses unofficial road building and forest fragmentation (Dourojeanni 2006). In addition, a set of hydroelectric dams is in the advanced planning stages on the Madeira River. The ‘Madeira Complex’ will cost at least US$10 billion and will affect several rivers and facilitate large-scale resource exports in the southwestern Amazon. Concerning climate change, 2005 marked a year of record drought in the Amazon, related to north–south Atlantic Ocean temperature differentials similar to those forecast by the Hadley land–climate model (other papers, this volume). This resulted in a prolonged dry season and created conditions for anthropogenic fires to escape control. The consequence in the southwestern Amazon was over 300 000 ha of primary forest burned and at least US$50 million in direct economic losses (Brown *et al*. 2006).

Road building and climate change threaten forests and livelihoods in the southwestern Amazon, a region of exceptional biological value. The southwestern Amazon exhibits exceptionally high biodiversity (Myers *et al*. 2000), especially in the Andes–Amazon transition, which according to the Hadley model may not be lost due to climate change during the twenty-first century (other papers, this volume). This possibility makes capacity building for environmental governance in the southwestern Amazon particularly important.

A key complication for environmental governance in the southwestern Amazon is that it is a tri-national frontier, called the MAP region, named after the three states that constitute the corridor where the Inter-Oceanic Highway is being paved: *M*adre de Dios (Peru), *A*cre (Brazil) and *P*ando (Bolivia). The MAP region encompasses roughly 300 000 km^2^ and a population of roughly 700 000, which includes many different stakeholders such as indigenous groups, forest extractivists, small farm colonists, large-scale ranchers, miners, logging firms and growing urban populations (Brown *et al*. 2002). Despite limited state capacity and considerable social diversity, the MAP region has birthed social movements with innovative policy proposals (Kainer *et al*. 2003). Trans-boundary challenges such as the Inter-Oceanic Highway and climate change prompted recognition of the need for cross-border environmental planning. This stimulated conversations across national borders among scientists, community leaders and government representatives (van Oosten 2004; Rioja 2005).

Such conversations fomented the emergence of the MAP Initiative, a grassroots social movement with a polycentric structure (www.map-amazonia.net). This structure is defined by a handful of key organizations on each side of the tri-national frontier which serve as nodes in a larger network of governmental and non-governmental organizations and communities. A key focus of the MAP Initiative is to collectively identify and advocate strategies to mitigate the negative impacts and capture the benefits of highway paving (Brown *et al*. 2002). By bringing together diverse stakeholders who depend on roads as well as local resources, the MAP Initiative seeks to avoid the pitfalls of relying on either governments or communities alone in seeking to improve road governance.

Simulations of future change in the MAP region indicate that with road paving and ‘business-as-usual’ land use, roughly 67% of forest cover and 40% of mammalian biodiversity there will be lost by 2050, though the losses would be reduced with improved governance (Soares-Filho *et al*. 2006). An overarching goal of the MAP Initiative has therefore been to build capacity across national borders for tri-national environmental governance. Consequently, the MAP Initiative has organized tri-national forums open to the public for presentations, dialogue and planning activities involving four key themes: economic development, environmental conservation, social equity and public policies (www.map-amazonia.net). This multifaceted array of themes allows for consideration of the many impacts of roads as well as climate change. Attendance at tri-national MAP forums grew from 25 individuals in 2000 to roughly 1200 in 2004, and peaked at 200 organizations represented in 2006 (www.map-amazonia.net).

This growth proceeded alongside the emergence of numerous ‘mini-MAP’ working groups with specific foci (www.map-amazonia.net). Mini-MAP working groups have engaged in many community outreach efforts. The ‘mini-MAP roads’ group organized a series of stakeholder workshops in municipalities along the Inter-Oceanic Highway on all three sides of the MAP frontier in order to envision the probable scenarios of future change due to road paving, and thereby initiate planning activities to avoid negative outcomes (Mendoza *et al*. in press). A ‘regional planning’ mini-MAP group has also emerged, with proposals for improved road governance along the Inter-Oceanic Highway corridor. This includes a ‘consultative letter’ for regional governments to sign in order to commit funds to implement MAP proposals for improved road governance (www.map-amazonia.net). This letter embodies a strategy with hallmarks of a hybrid governance model: participatory deliberations to formulate proposals for resource governance in the presence of a large-scale infrastructure project, presented to governments for approval and commitment of funds, in return for compliance by local stakeholders.

As the MAP Initiative has engaged local stakeholders, it has also sought to scale up its policy impact via increasing contacts with governments on all levels (www.map-amazonia.net). Several state and municipal governments in the MAP region have officially expressed support for the MAP Initiative by inviting and/or implementing policy input from organizers, including for road governance. MAP Initiative organizers have also met with—and been officially recognized by—the Ministries of Foreign Relations in Brazil and Peru (www.map-amazonia.net). MAP Initiative organizers have also met with international lending agencies to discuss strategies for securing funds to support cross-boundary environmental governance.

The growth of the MAP Initiative on tenuous budgets, and the scale mismatch between MAP Initiative, the Inter-Oceanic Highway and climate change, led to recognition of the need for capacity building of the MAP Initiative itself. This prompted negotiations with governments and funding agencies for resources to accelerate cross-border activities for tri-national environmental governance in the MAP region.

One result was to secure funds from the US Agency for International Development through the Amazon Basin Conservation Initiative (USAID 2005; ABCI 2007) to support the ‘MAP’ Consortium. The MAP Consortium is organized in two parts, one for Brazil and one for Peru and Bolivia, and encompasses eight institutions involved in the MAP Initiative from Bolivia, Brazil, Peru and the US, including NGOs, universities and a government agency. This line-up affords contacts to both governments and civil society, and embodies a strategy to enable a hybrid governance model that reflects the experience of the MAP Initiative. The goal of the MAP Consortium is to increase the capacity of the MAP Initiative for governance of trans-boundary watersheds and sustainable development planning in trans-boundary road corridors. Both will proceed via coordinated data collection and monitoring, paired with stakeholder workshops to discuss the findings and formulate likely scenarios of future change, which then serve as a basis for participatory planning with government agencies. A key requirement for such planning efforts to have lasting impacts is to combine them with capacity-building activities, including environmental education of future leaders, as well as cross-boundary exchanges among stakeholders and government representatives to improve coordination of governance initiatives.

The underlying strategy of the MAP Consortium is to premise trans-boundary environmental governance on sustaining an autonomous, polycentric structure that does not rely on a single centralized authority such as a government, or on a fully decentralized, uncoordinated network such as a set of local communities (Ostrom 2005). By retaining flexibility while ensuring coordination, the MAP Consortium constitutes a structure for collaborative environmental governance that can manage itself adaptively in order to respond quickly to rapid changes (Wollenberg *et al*. 2007). Given the experience of the MAP Initiative, the MAP Consortium seeks to leverage past social learning through the complementary strengths of its member NGOs, universities and government agencies in a process of ongoing collective learning (Keen *et al*. 2005). Theoretically, this will allow the MAP Consortium to accelerate planning for environmental governance of trans-boundary watersheds and road corridors in the face of future changes, such as that due to climate change. However, the MAP Initiative and the MAP Consortium are in effect testing hypotheses. It remains to be seen if this experiment in environmental governance will prove sufficient for the task of conserving Amazon forests and the livelihoods of people who depend on them.
